# Analysis of the MUII-plus mentorship programme: reflections of Fellows’ experiences and lessons for other programmes

**DOI:** 10.12688/aasopenres.13091.1

**Published:** 2020-08-10

**Authors:** Irene Andia Biraro, Emmanuella Driciru, Rehema Namaganda, Fiona Luboga, Charles Kato Drago, Anne Wajja, Brenda Okech, Mary Gorrethy N. Mboowa, Raymond Muganyizi, Moses Kizza, Stephen Cose, Victoria Diana Bukirwa, Damalie Nakanjako, Alison M. Elliott

**Affiliations:** 1 Department of Medicine, School of Medicine, College of Health Sciences, Makerere University, Kampala, Uganda; 2 Immunomodulation and Vaccines Programme, Medical Research Council/Uganda Virus Research Institute and London School of Hygiene and Tropical Medicine Uganda Research Unit, Entebbe, Uganda; 3 Makerere University-UVRI Center of Excellence in Infections and Immunity, Entebbe, Uganda; 4 Department of Child Health and Development Center, School of Medicine, College of Health Sciences, Makerere University, Kampala, Uganda; 5 College of Veterinary Medicine, Animal Resources and Biosecurity, Makerere University, Kampala, Uganda; 6 UVRI - IAVI, Entebbe, Uganda; 7 Clinical Research Department, London School of Hygiene & Tropical Medicine, London, UK

**Keywords:** Mentorship, MUII-plus, SWOT, Research, academic careers, Africa

## Abstract

**Background:** The MUII mentorship programme began 11 years ago with a successful group mentorship model. Over the years, the programme has evolved and is presently anchored on the “GROW” approach. This model allows individuals to: set Goals (What I want?); Reflect (Where am I now?); think of Options (What can I do?); What to implement (my actions?). It is intended to help fellows (current, honorary, alumni) herein referred to as mentees achieve their short, medium, and long-term research, career and professional goals.

**Methods:** A mixed methods study combining a cross-sectional survey, one focus group discussion and 11 in-depth key informant interviews were carried out between November 2018 and January 2019 to 1) assess the status of the mentorship programme, 2) perform a strength weakness opportunity and threats (SWOT) analysis, and 3) identify factors relevant for sustainability.

**Results:** An open invitation was made to 52 fellows to participate in the survey, and 23 responded. Among respondents, the largest proportions were male [70% (16/23)], and PhD fellows [35% (8/23)]. The respondents rated the fellowship experience as excellent [65% (15/23)], and most [78% (18/23)] revealed they had benefitted greatly from the programme. The SWOT analysis revealed outstanding strengths of having regular fellows’ meetings for peer support, and availability of international collaborations, linkages and exposure. Opportunities identified included large pool of mentees within MUII-plus and evidence of fellows taking up leadership positions. The biggest threat to the mentorship programme was the busy schedule of mentors.

**Conclusions:** The MUII-plus mentorship programme has strong potential to offer research and career mentorship to its fellows. To promote sustainability of the programme, there is a need for innovative ways to engage mentors; such as digital platforms (e-mentorship) for greater mentor-mentee interactions.

## Background

Mentorship in science, research and capacity building programmes is essential to promote personal and professional advancement especially in low- and middle-income countries (LMIC) such as Uganda ^1, 2^. Formal mentorship programmes in capacity building or tertiary education offer objective strategies for transfer of knowledge or skills by the mentor to the mentee ^3^. Although formal mentorship programmes have positive attributes ^4^, they face several challenges such as difficulty in achieving the perfect mentee–mentor matches, effective or sustainable mentoring through mutual respect and trust, and overlap of mentor roles ^5, 6^.

The Makerere University Research Training Programme in Infection and Immunity (MUII) programme is a collaborative capacity-building and research programme which has been supporting excellence in infection and immunity in Uganda for over 10 years. MUII has attracted bright young Ugandans to develop careers in infection and immunity research. Up to 68 research fellowships have been offered, including three group leader, 15 post-doctoral (post-doc), 27 PhD and 23 Masters fellowships. These trainees have been mentored by MUII academic staff from Makerere University and Uganda Virus Research Institute, as well as faculty from MUII collaborating institutions such as University of Cambridge and the London School of Hygiene & Tropical Medicine.

One of the hallmarks of the MUII programme during the first five years was the successful informal group mentorship sessions among the MUII fellows. In addition, Masters fellows were formally paired with more senior fellows who supported their progress. During MUII-plus, a formal mentorship team was established to coordinate mentorship activities in the programme. The mentorship programme adopted the GROW model approach for ono-to-one mentoring relationships. This approach allows individuals to set short, medium and long term Goals (What do I want?); Reflect (Where am I now?); think of Options (What can I do?); and decide What to implement (proposed way forward actions?). Currently, mentees and mentors post their profiles on the MUII-plus mentorship website and mentees are given a chance to choose their mentors. This is a good practice because it promotes mutual respect. However, because there are more mentees than mentors, the mentee to mentor ratio is usually about 3:1.

There is local anecdotal evidence of the usefulness of group mentorship in facilitating progress of the fellows in their training under the MUII-plus programme. However, this evidence has neither been documented nor disseminated because of lack of proper data to back up the observation. In addition, the number of mentees outweighs the critical numbers of mentors available to provide mentorship. Many educational and capacity-building programmes suffer a similar fate ^7, 8^. As a consequence, many mentorship programmes have an ad hoc approach to mentorship focusing mainly on either peer or individual mentoring relationships and professional development. Many also lack essential toolkits that provide practical guidance and assessment structures for orientating or directing both mentees and mentors in such programmes ^2, 7, 9^.

The MUII-plus mentorship programme therefore carried out a study to 1) document the mentorship situation of the programme based on the mentorship experiences of the fellows; 2) assess the Strengths Weaknesses Opportunities Threats (SWOT) of the mentorship programme to facilitate the designing of a framework against which the programme would be monitored and evaluated; 3) provide a platform that would allow for constant quality improvement assessments based on the principles of Plan, Do, Study, Act (PDSA); and 4) determine factors that would lead to a sustainable mentorship programme.

## Methods

A mixed-methods cross-sectional study was conducted between November 2018 and January 2019 in Kampala and Entebbe, Uganda, among a cohort of current and former MUII-plus fellows. An open invitation was made to all current and former MUII fellows to participate in the study. This approach is most ideal for this assessment since it involves concurrent and systematic integration of both quantitative and qualitative data, permitting a more complete and synergistic utilization of data from a range of sources, during data collection, analysis, and discussion. A strategic element of the survey to promote ownership of the survey findings and facilitation of their utilisation, was to involve the programme leadership, administrative staff and alumni.

### Quantitative data collection

The survey used structured online questionnaires to collect primary data (available as *Extended data* ^10^). The survey data was used to assess the target groups’ views on the mentorship programme and ideas on how it can be improved. The survey was sent by email to respondents that included masters and PhD students, mentors and leaders of the MUII programme. The survey data included age, gender, role of respondent in the MUII-plus, duration on the MUII programme, rating experience in the mentorship programme, suggested areas of improvement of the mentorship programme, areas of knowledge or skill transfer, areas of support the respondent can render to the mentorship programme.

### Qualitative data collection

Qualitative data was collected using key informant in-depth interviews (IDIs) and focus group discussions (FGDs). A telephone log of the 52 active MUII-plus fellows, five MUII-plus administrators and five MUII-plus alumni was provided to the study researcher, who later called up all the participants requesting to be interviewed either as part of FGDs or IDIs respectively. Study eligibility and enrolment was based on availability to participate during the approved study period. A single FGD was conducted with eight PhD fellows, of whom three were female and five male. A further 12 IDIs were carried out with two MUII-plus top administration, two group leaders, and four masters’ fellows, one post-doc fellow, and three MUII-plus alumni. Both the FGD and IDI guides (available as *Extended data* ^10^ explored themes related to the MUII-plus mentorship programme and fellow’s experiences, and SWOT assessment of the programme. All interviews were conducted face to face at the workplace except for one that was conducted via Skype, and were all audio-recorded. The IDIs took about 15–40 minutes while the FGD took approximately one hour. The IDIs took place at the different workplaces of the interviewees, while the FDG took place at the MUII-plus Center of Research Excellence building in Entebbe, Uganda. There were five males and seven female respondents interviewed in the study. Field notes were made during the interviews, especially for the FGD. Interviews ended when all respondents started providing similar responses at data saturation. At the end of each interview, the researcher would re-cap the different discussion points represented by the respondents and general consensus obtained and where needed, corrections made. After the interviews, the transcripts were transcribed verbatim. One member of the administration, who was new to the MUII-plus programme, felt they were unable to adequately provide key information needed and therefore this interview, which took only 5 minutes, was excluded from the analysis.

### Data analysis and presentation

Quantitative data from the online structured survey was cleaned and manually edited for any irregularities. The data captured was exported to SPSS version 11.0 (Statistical Package for Social Sciences) for more detailed analysis. The data was analysed to demonstrate frequency and percentages of the collected variables. Simple frequency tables were used to report the analysed data. Qualitative data was analysed following thematic framework analysis ^11^. This involved reading and rereading transcripts, summarizing them using matrix, categorizing and identifying themes. One data collector coded the data and this was exported into ATLAS.ti software version 8.3 for further analysis. The main theme was the mentoring programme and the sub-themes were focused on the SWOT analysis. Data was presented in thick description as text and privileged the voices of the interviewees using relevant representative quotes.

### Ethical considerations

The study was approved by the School of Medicine Research and Ethics Committee, Makerere University College of Health Science (REC number 2018-171) and the Uganda National Council for Science and Technology (SS-4921). Online written consent was sought for the fellows before they could proceed to answer the survey questions. For the qualitative interviews, verbal and written consent was sought from all participants involved in the study.

## Results

### Respondent characteristics

Among 52 fellows who had been supported by MUII by the time of the survey, a total of 24 respondents participated. One participant consented and started filling out the online survey but did not complete it by the end of three months study period, and was therefore excluded from the study. Our response rate was 44% (23/52). Respondent characteristics are shown in Table 1. The group leader was also a member of the MUII-plus executive committee and three of the doctoral fellows were honorary MUII-plus fellows (the primary funding for their fellowships was not directly under MUII-plus but through MUII-plus collaborations). Individual-level responses to the survey are available as *Underlying data* ^12^.

**Table 1.** Respondent characteristics. Table shows the characteristics of the 23 respondents that participated in the quantitative survey.

Variable	Level	n (%) n = 23
Gender	Male Female	16 (70) 7 (30)
Age (years)	20 – 30 31 – 40 > 40	9 (39) 10 (44) 4 (17)
Cadre	Group leader Administrative staff Intern fellow Master fellow Doctoral fellow Postdoctoral fellow	1 (4) 1 (4) 4 (17) 4 (17) 11 (49) 2 (9)
Role	Mentee Mentor Both mentee and mentor Not applicable	15 (66) 1 (4) 4 (17) 3 (13)
Duration in programme (years)	5 – 10 2 – 5 1 – 2 < 1	2 10 9 2

### Knowledge transfer and mentoring experience

The respondents were asked about their previous mentorship experience and how this contributed to their career progress through the different forms of knowledge transfer. Overall, 65% (15/23) of respondents reported an excellent experience, 22% (5/23) a good experience, 13% (3/23) a fair experience, and none reported a poor experience which was a good reflection on the mentorship programme. In total, 18 participants felt they benefited from the mentorship programme (78%), two (9%) were not sure about any benefits, while three respondents (13%) felt they had not benefitted from the mentorship programme. Of those that reported no benefit, one was staff, one an honorary doctoral fellow and the other a MUII-plus doctoral fellow. The respondents reported different types of benefits, which included help with applying for additional grants, proposal writing, presenting PhD papers, designing and writing Masters’ projects, identification of trainings to attend, introduction to key persons or collaborators, and motivation to go for further studies. Specific areas that knowledge or skills were transferred are shown in Table 2. In addition, 74% (17/23) of all the participants felt that mentorship contributed to their career enhancement. Table 3 describes the different career enhancement theme areas that the respondents attributed to mentorship.

**Table 2.** Areas of knowledge or skill transfer. Table shows the different mentorship output area reported by the 23 survey respondents where mentors transferred knowledge or skills. These were multiple response options.

Area	Skill transferred	Frequency
Publications	Manuscript writing Manuscript submission Response to reviewers None	11 1 1 9
Presentations	Slide preparations Presentation practice None	14 13 2
Grants	Grant call alerts Grant idea brainstorming Grant writing Grant submission Grant review process None	10 4 5 1 2 7
Research Projects	Research ideas Research proposal writing Project work Thesis work Thesis defense None	9 14 9 9 4 1
Community Engagement	Media event Research project sensitisation Dissemination of research findings None	5 3 8 9
Fellowships or Job Applications	Fellowship or job alerts Writing a personal statement Writing a curriculum vitae Writing an application or cover letter Practice for an interview None	10 5 2 2 7 7

**Table 3.** Ways how the mentorship programme has contributed to the careers of respondents. Table showing multiple areas of mentorship outcomes from multiple responses from the 23 survey respondents.

Theme	Frequency
Exposure to new research networks, collaborations, perspectives, ideas and approaches	13
Increased confidence and motivation for research work and science	16
Improved research leadership and management skills	7
Promoted professional development	10
Not applicable	1

### Mentorship programme

Understanding the strengths (S) and weaknesses (W) of, and the opportunities (O) and threats (T) to the mentorship programme was very important to the understanding of its current and future capacities to deliver on its intended goals. The survey aimed at finding out the above using the SWOT approach in the key informant IDIs and FGDs. A summary of the SWOT findings are summarised in Table 4. Of the interview respondents, one was from top administration and three were alumni; of which one was a former MUII PhD fellow and two former MUII post-doc fellows. The post-doc fellow interviewed was formerly a MUII Masters and MUII-plus PhD fellow. Of the two group leaders that participated in the IDIs, one was a former MUII PhD and post-doc fellow while the other was a former MUII post-doc fellow. Three of the masters’ respondents were from the Masters’ of Medicine (MMED) scheme.

**Table 4.** Summary of the SWOT analysis. SWOT analysis was carried out as part of the study to assess the MUII-plus mentorship programme using key informant in-depth interviews.

Strength	Weaknesses	Opportunities	Threats
1. Strong / new leadership supporting the mentorship programme 2. Well trained scientists to act as mentors 3. Well-equipped laboratory to allow research growth 4. Availability of locally based mentors. 5. Regular fellows meetings from which reviews are made and provides platform for group mentorship 6. The attitude of people supporting each other. General supportive attitude. 7. Regular follow ups by MUII plus administration and internal monitoring as a form of accountability mentorship 8. Access to international mentors 9. Resource base for persons that operate as a family 10. Collaboration and linkages. 11. Strong coordination and reputation profile of MUII plus	1. Management setting reliance on the institution framework 2. Unclear sustainability plan of the mentorship programme 3. Busy schedules of the mentors 4. The alumni are less involved in the programme. They need to be kept around 5. Scholars are not all at same campus like with some Delta programmes	1. Availability of many students to be mentored 2. Availability of mentors from different fields such as TB experts, Gynaecologists, Immunologists 3. Future funding for post- docs 4. Some fellows taking up leadership positions 5. A lot of research still needs to be done 6. Enabling environment for training or being trained by young men and women 7. Affiliation with other institutions and people which makes mentorship easy	1. The procurement process is not direct and has severe delays which is a threat to the dynamics of research careers 2. Future funding uncertainty 3. Busy schedule of the mentors 4. Disappearance of the alumni. There is no continuity

***Strengths.*** The programme was rated highly by the alumni and mentees who were interviewed for the study. There was agreement that the programme is very beneficial both to the mentors and mentees. Voices from the alumni strongly attach high regard for the programme and here below are some of the identified strengths of the programme.

**Allows knowledge transfer and academic interaction**: The mentees revealed that the architecture of the programme allows for knowledge transfer through interactions between mentees at various levels for example those pursuing doctorates, masters and post-doc fellows. This makes the fellowship programme very enriching and relevant to the mentees.

*“…fact that they have several categories of layers of PhDs, post-docs, masters all interacting and learning from each other in presence of senior researchers. I think it is a tailored approach of mentoring. So it is peer mentoring within the same levels but also without…”*-Alumnus 1- KI

The programme gives an experience of interaction between the young scientists and senior scientists which facilitates knowledge transfer. This is an indication that the programme is well designed to deliver on its objective of producing highly skilled and knowledgeable scientists. There is evidence in the data that the mentors on the MUII-plus mentorship programme offer their best to the mentees, resulting into long life relationships that guide the mentees through their professional and academic endeavours. Also, the mentees praised the mentors for always treating them with courtesy even on issues beyond the scope of the mentorship programme. The study further revealed that the group mentorship programme offers team work to its members from which they draw energy to attempt to achieve a wide range of goals. For example, one of the members of the FGD shared that the mentors have been guiding them both face to face, and online as they went about their academic study.

*“The mentor has been really guiding me and over the last one year while I was away she was also away and we happened to be in the same state. So when the mentorship continued, we were having either face to face meetings or phone calls. She even visited me at my University where I was---” -* **FGD R4**

***Weaknesses.*** In spite of the various programme strengths and goodwill from both the mentors and the mentees, the mentorship programme exhibits a number of weaknesses which affects its success.

**Busy schedule of mentors**: Voices extracts from the alumni show that the programme is undermined by the busy schedules of the mentors and lack of commitment by some, which leaves the mentees at the mercy of individual initiatives. Many of the mentees that were paired with mentors outside their field of study struggled to appreciate the mentor’s inputs.

*“Some mentors are not committed, these guys don’t have the same enthusiasm, so if I call him, he says I will be there and then he doesn’t show up”* - **FGD-R7**

***Opportunities.*** The study identified the following as key opportunities which the programme can continue to harness and promote for its success.

**Pool of students available**: The programme receives a pool of students and academic staff from Makerere University every year. This presents an opportunity to have a pool of mentees and mentors. One of the KIs viewed this as a big opportunity to help the mentorship programme remain active.

**Positive attitude of mentees**: The FGD further identified the positive attitude of the mentees which makes them strongly associate with the mentorship programme and their willingness to make it succeed during their time with MUII-plus.

***Threats.*** The mentorship programme faces some challenges or threats, though some respondents indicated that these were not that big to pose a serious danger to the programme.

**Busy mentors**: There was a general agreement among mentors and alumni that there is difficulty in identifying committed mentors and motivating them to stay on the programme and accord enough time to the mentees. There is an need to ensure that mentors accord more time to mentees because it was clear that mentors scarcely accord time to the mentees and often at times the mentees had to figure out ways to address their challenges themselves.

**Lack of motivation**: This may be related to lack of motivation of the mentors beyond the satisfaction of successfully mentoring the mentees. During the FGD with the students, they expressed the view that mentors may not be motivated enough to focus on the mentorship programme. Students were divided on what kind of motivation that should be given to a mentor: some said that a mentor shouldn’t be motivated in terms of financial or material benefits, but should have the natural desire to provide mentorship, while others were strongly convinced that as long as mentors are not financially motivated, they will not give time and commitment to this cause.

***Other achievements.*** Other important additional themes related to mentorship emerged from the interviews as reported below.

**Role modelling**: There was a general consensus by all the interviewees that the mentorship programme has registered a success from all perspectives (mentors, mentees and funders). However, the nature of achievement may vary from tangible and no tangible programme achievements. For example, a mentor may achieve, and also be motivated by seeing their mentees succeed in their academic activities while the mentee maybe motivated by successfully finishing their academic programmes and are awarded their masters, PhDs and post-docs.

**Good reputation of MUII-plus**: The programme has been cited as one that has produced very good scientists that have gone ahead to impact society in various ways. The programme boasts of being a hub through which a number of fellows have had opportunities to carry out cutting-edge research which has helped them earn advanced academic degrees.

*“I think it has been really exciting to see people’s careers develop. From the first group of fellows we had 4 PhDs and 2 post-docs from whom now we have a Dean, deputy Dean, 2 head of departments, and 2 others who are also becoming increasingly senior academics. So many of them, like the PhD fellows, we have supported them from an initial ground and we have been able to provide a second round of funding to support some of them again, so it’s really helped in career progression…”* -Executive 1-KI

**Downstream effect:** The mentorship has yielded further capacity building for the University departments where mentees and beneficiaries come from. This has been achieved by providing a platform where various scholars from various university colleges meet as they work on various doctoral and non-doctoral research studies.

**Community engagement**: The recent inclusion of community engagement to the MUII-plus programme was cited as an opportunity for scientists to disseminate their findings to the people who are supposed to benefit from them. Voice extracts from the focus group discussions show that the community approach bridges the gaps between the scientists and the final recipients of the research findings

*“….about this aspect of community engagement which we started recently, I liked it very much because its good but also maybe they could push it a little bit forward to strengthen it like most of the people are doing work which is related to health issues but who is the final consumer, let alone producing paper work here. The final consumers, those are the people, the community”*—FGD-R8

### Factors leading to a sustainable and successful mentorship programme

The survey aimed at finding out the factors which the various respondents thought would lead to the success of the MUII-plus mentorship programme building from the milestones of the previous informal group mentorship programme. The majority of the participants indicated the need to create more opportunities for mentee–mentor interactions. Table 5 discusses the different areas for improvement and sustainability of the mentorship programme.

**Table 5.** Areas of improvement for the mentorship programme.

Area of improvement	Frequency
Carry out more awareness messages about the mentorship programme	11
Improve leadership of the mentorship programme	4
Create more platforms for mentor – mentee encounters	16
Conduct more training for mentors	8

**Involvement of alumni**: Involving more alumni was cited as a major point for sustainability of the mentorship programme. Also, the need to involve more senior researchers or scientists especially from University teaching staff and research communities was noted.

*“I don’t know how you can get senior mentors involved more. I don’t know if there is like a reward system or like recognition for what they do, that will encourage senior mentors to take on mentorship”* -Alumnus 1-KI

**Reward mentors**: In line with the above suggestions, the survey found out that there is need to reward mentors for their time in order to secure their buy in into the programme especially giving more time to the mentees. The rewards do not necessarily need to be monetary.

*“I’m also of a view that these mentors should be rewarded*. *(F: For motivation) Yes, because if you reward these people definitely they will participate more”* -FGD-R2

*“And also applauding them let’s say at an AGM and then they say so and so we appreciate you for being a good mentor, such things”*—FGD-R3

**Clarification on roles and expectations**: The study further revealed that respondents see a need to streamline the conduct of business in the mentorship programme, wherein documentation detailing key responsibilities of mentors and mentees, structure of the process and targets to guide evaluation need to be laid out to avoid confusion. And that the programme should appreciate the other aspects of the human being especially the social aspects which mentors should be cognizant about when handling the affairs of the mentees.

De-identified transcripts from KIIs and FGDs are available as *Underlying data* ^10^, 13.

## Discussion

Mentorship is an integral part of capacity programmes such as MUII-plus as it promotes research interests, especially in neglected fields in LMIC countries such as immunology. The use of SWOT analyses and baseline surveys are always recommended for programmes, including mentorship programmes, to provide data that can assess current systems, and also highlight areas for future improvement ^14^. Our survey had a low response rate similar to some reported surveys ^15^. The low level of response by fellows could have been attributed to the use of online methods of data collection which could have been challenging for some non-techy fellows. Despite the MUII-plus programme being more gender balanced, most of the respondents in this study were male. A majority of the respondents were mentees, which might have skewed the findings and their interpretations thereafter.

The study revealed that most of the respondents found the mentorship experience excellent and had benefitted from it through various channels of knowledge transfer. They also mentioned that the group mentorship sessions allowed for social interaction ^16^ and enhanced learning as they were able to meet with fellow mentees or mentors that are carrying out a variety of research. This also gives the fellows a sense of belonging which inspires their research productivity. Mentee satisfaction ^15, 17^ is usually derived from different sources including availability of funding and opportunity to interact with likeminded scientists ^18^, which has accelerated some of the respondents’ journey to academic success. Such group mentorship helps fellows receive a clear focus and roadmap in their training ^19^. The need for continuation of group mentorship was echoed during the interviews. The fellow’s meetings offer a platform for group advice by the few mentors present, and also aids research accountability and improves presentation skills. Mentees share experiences and receive research or career encouragement. This also prepares them to become future leaders and mentors. Group mentorship is still a recognised approach in several capacity building programmes, especially where the number of mentees in need of mentorship is high compared to mentors, as in the case of the MUII-plus mentorship programme ^20, 21^. Such group mentorship also helps in achieving high completion rates of mentees ^8, 22^. There are skill transfers and advisory support by more senior fellows to junior colleagues during the group mentorship sessions at the fellows’ meetings as in similar cases across universities in Uganda ^23^. In addition, mentorship by local mentors in MUII-plus helps with navigation of institutional administrative requirements, helping the mentees to settle faster. Some of the challenges with this approach can be the risk of provision of novice mentorship, and lack of proper assessment or documentation.

Good mentors must intend to be good communicators and grow the mentees. While good mentees must have an adaptable character, be self-directed and recognise generational differences ^24, 25^. The study highlighted that the MUII-plus mentorship programme provided an enabling and safe environment without inappropriate gender conflicts ^16, 26^. There was adequate research support to develop research ideas or problem solving, and guidance ^25^. This increased the level of the student’s academic and professional productivity mainly measured by retention in the programme, number of publications and grants awarded ^1^ ^27, 28^. Therefore, this opens to us a window with more clarity that the MUII mentorship programme has had a cascade of benefits which have in one way impacted the lives of the fellows. However, Zhang *et al* ^29^. assert that for a much better experience, the mentorship approaches should be well thought out, planned and executed in order to have the best outcomes. In this vain, they propose that rigorous mentor selection and adequate training, identifying potential barriers such as time constraints and scheduling limitations should be taken into consideration during implementation of a mentorship programme.

Further still, the different mentorship styles should also be illustrated in mentorship programmes. There are those that promote mentee empowerment and encourage reflective practices. Some that are geared towards checking or observations of milestones, and others that are more directing or authoritative to get tasks done ^30^. Usually a triangulation of styles is often used in most mentorship experiences. Also, good mentoring relationships should be based on the Martin Buber’s theory of “I towards Thou”, where the mentor grows the mentee irrespective of recognition or reward as opposed to “I towards It” which seeks only tangible gains from the relationship. If a mentee–mentor pairing is not aligned, it leads to ineffective mentoring relationships and reduces mutual trust. However, this whole process is difficult to get right ^5, 28, 31^. The mentees, however, didn’t report any mentor mismatches or mentor malpractice during their experience. Possibly the lack of set tracking systems for accountability or assessment might have affected the mentee–mentor relationships and outcomes. How to balance the nature of mentee–mentor relationship can also be tricky with merging of boundaries between formal mentor role as the advisor with the informal role as the friend ^16^. It is still important to engage both roles for effective mentorship without crossing ethical and professional boundaries. The MUII-plus programme has a policy on gender and diversity that supports vulnerable mentees and mentors. It is also advised that mentees should have a diversity of mentors ^25^ to be able to get a wholesome mentoring experience.

The weakness and threat of having busy mentors in the MUII-plus mentorship programme could be as a result of mentors having overlapping roles as advisors, advocates, supervisors and teachers, as well as role models. Multiple roles might result into role conflict or confusion reducing effective mentorship in terms of performance and productivity, which is a common occurrence in mentorship programmes ^4– 8, 28, 30, 31^. Mentors are bound to be busy as they grow in their careers. Mentors’ busy schedules has been reported in other capacity-building programmes. Many mentors are involved in several academic activities, including research, teaching, administration and (for some) clinical work. The various activities play a big role in the busy lives of mentors. Many mentors lack protected time dedicated to mentorship. In addition, some that are involved in mentorship are not recognised for their efforts ^7, 22^. On the positive side, both mentees and the few mentors had a “pay it forward” attitude towards the mentoring process ^16, 22^. This is a sense of paying back to mentorship as a benefactor or recipient of good mentorship that led to personal or professional success.

It is crucial for mentors in capacity-building programmes to have the necessary competencies ^9^. This study, however, was not able to access such competencies. MUII-plus provides a holistic mentorship experience for most fellows that addresses their welfare, drives professional progress and promotes leadership skill. This is evidenced by many fellows taking up leadership positions. However, many of the current mentors are in their early to mid-careers and may be unable to provide holistic mentorship because of their work life challenges. They too usually lack research mentorship ^33^. Work life pressure mostly among mentors is a documented challenge ^34^. MUII-plus collaborators have provided the much-needed additional mentors for the mentorship programme, easing the load on the few local mentors. This has been reported as an advantage in other programmes ^35^. The slogan “You travel faster alone, but further together” has been one of the hidden MUII-plus mentorship mottos. It is necessary for mentorship programmes in LMIC countries to continually network with more established institutions in high-income countries for research mentorship support ^36^. The use of programme alumni as mentors was voiced as an opportunity to improve the MUII-plus mentorship programme and also promote sustainability of the programme. There is evidence that this is an effective approach ^27^. Unlike other programmes, in the MUII-plus programme, availability of physical space and laboratory services were highlighted as strengths.

Mentoring is a two-way relationship. The best results are achieved when mentors use their experiences to guide mentees ^24, 31^, for collaborative learning ^18^. In a formal mentorship programme with longitudinal relationships that last more than a month, there is need for mentee-mentor agreements to formalise the relationships and clarify on roles and expectations ^37^. In our study, the known threat of cultural, social and gender influences on mentee–mentor experiences was absent ^2^. Many mentees were able to identify “mentor role model figures” that contributed a lot to their personal and professional growth. For continuity and sustainability of capacity building programmes, it is crucial that mentorship ensures transfer of knowledge and skills down generations ^3^. In order to get the most out of the mentors, a structured system is needed ^25^. For the success of the MUII-plus mentorship programme, the respondents suggested that the current leadership should involve more senior researchers and scientists as well as alumni to foster continuity to the programme. The MUII-plus mentorship programme has also set up an online system to further structure the programme; however, this system needs to be studied continuously. In addition, the team developed a shared plan that mentees and mentors have to submit at the beginning of their mentorship relationship for accountability and documentation of activities.

Our biggest limitation in this study was the risk of selection bias. There was an open invitation sent to the fellows to participate in the online survey, and possibly those that responded were the ones that probably appreciated the programme most or benefitted from mentorship. This also needs to be deciphered further as the respondents were mostly male and yet the program is gender balanced. There may be some underlying challenges for females which need to be uncovered and rectified. Another study limitation was that the survey questions might have been skewed more towards reflecting mentee experiences than mentor experiences. This was made worse by the low participation of mentors. It would have been important to know what the difficulties where for the mentors and if there was a way to make the mentorship relationship a win–win for both the mentor and the mentee. Regarding the qualitative interviews, there was a balanced selection across groups. Out of the 11 interviewees, seven have been part of MUII programme since inception, and therefore had institutional memory to ably respond to the questions. There might be some slight bias with the alumni since the formal MUII-plus mentorship programme started after they left and therefore might have introduced some social desirability bias. In addition, the masters’ fellows interviewed were all from the MMED scheme, and their responses might not have been representative of all other science programmes in MUII-plus. The interviews were conducted by one researcher, which could have introduced some confirmation or wording bias. However, this was managed by the study team reviewing and agreeing on the findings. Also, the SWOT analysis could have assessed the mentorship programme at three levels namely institutional, mentee and mentor levels for better representation of the key areas.

## Conclusion

The mentorship programme is beneficial to all the stakeholders and it is important for the leadership to know that the respondents believe in the programme and are ready to accord it all the necessary support. The programme has more strengths and opportunities than weaknesses and threats. However, the programme faces a challenge of attracting and retaining mentees and mentors who are committed to the programme. The most exciting discovery from the survey is that the mentorship programme still enjoys support of both the mentors, mentees and alumni and majority are ready to support its agenda. This buttresses the programme and magnifies its strengths and opportunities in comparison to its weaknesses and threats. The overall MUII-plus programme has and is committed to support the implementation of the mentorship programme, which is a big boost towards a sustainable and successful programme. The findings from this study can be used by other mentorship programmes in capacity building, as a benchmark and also for quality improvement by the MUII-plus mentorship team for better performance. Going forward, the mentorship team recommends the following:

1. Innovate ways of engaging both mentees and mentors, including use of new platforms or solutions such as digital mentorship (e-mentorship) ^16, 38^. However, limitations such as loss of personal or emotional bonding need to be noted.

2. Employ several opportunities for speed mentorship to avoid fatigue from long relationships ^39^.

3. Consideration of a new contemporary mentorship approach known as reverse mentorship. This reverses the top down directional mentorship to bottom up approach where the mentor is less experienced, and mentee more experienced. This is an upcoming approach in this era of technology, especially for biomedical or laboratory-based research mentorship because of its advantages of narrowing generation gaps and opportunities for shorter mentoring relationships ^40, 41^.

4. Promotion of a hybrid mentoring system for the MUII-plus mentorship programme that supplements the formal structural longitudinal relations with the already successful informal group mentorship sessions during the quarterly fellows meetings.

5. MUII-plus online, web-based toolkit to orient the fellows and provide guidance on the mentorship programme. The digital toolkit will also include check lists that will assess the different mentoring milestones. These will also be used for electronic activity calendars to help track activities and mentoring encounters ^16, 37^.

6. Establish coach mentors or certified mentors ^42, 43^ as a form of recognition for mentors.

7. Create frequent channels for messaging as a platform for feedback. Additionally, other forms of communication channels such as Zoom or Skype could be used for meetings ^25^.

## Data availability

### Underlying data

Figshare: Mentorship questionnaire responses.xlsm. https://doi.org/10.6084/m9.figshare.12624992.v2 ^12^.

This project contains the responses of each participant to each of the survey questions.

Figshare: Mentorship Programme Study Transcripts.pdf. https://doi.org/10.6084/m9.figshare.12624974.v2 ^44^.

This project contains the de-ide notified transcripts from in-depth interviews.

Figshare: FGD with MUII PhD students.pdf. https://doi.org/10.6084/m9.figshare.12624968.v2 ^13^.

This project contains the de-identified transcript from the focus group discussion.

### Extended data

Figshare: Study quantative and qualitative tools.pdf. https://doi.org/10.6084/m9.figshare.12624113.v2 ^10^.

This file contains a blank copy of the survey and the guides for key informant interviews and focus group discussions.

Data are available under the terms of the Creative Commons Zero "No rights reserved" data waiver (CC0 1.0 Public domain dedication).
