# Supplementary material for: Analysis of the MUII-plus mentorship programme: reflections of Fellows’ experiences and lessons for other programmes
Source: AAS Open Res. 2021 Mar 16;3:37. Originally published 2020 Aug 10. [Version 2] doi: 10.12688/aasopenres.13091.2 (PMC11109704; doi:10.12688/aasopenres.13091.2)
Supplement: Peer review history

# Reviewer report: Reviewer response for version 2

**DOI:** 10.21956/aasopenres.14323.r30707

**Published:** 2024-05-10

**Authors:** Daniel Patrick Balestrini

**Affiliations:** 1 University of Regensburg, Regensburg, Germany

I congratulate the research team on their research and their manuscript,

 Due to time constraints, I was only able to read the main manuscript. I did not have time to review the tables, the supplementary materials, or the bibliography. However,

 Most of my observations refer to mechanical or grammatical aspects.

 In some cases, I raise more fundamental questions. These should be addressed by the authors prior to publication via changes or an explanation.

 Should the editor oblige the authors to reply to my comments: I don't think responses are needed from the authors on any of the mechanical or grammatical points.

 The editor should make final determinations regarding the authors' changes.

 Finally, I have one remark for the editor and the journal: I did my best to review the article within the amount of time I had to do so. I stated in my comment that I only had time to review the main manuscript (no review of the tables, the bibliography, or the supplementary materials). In order to submit my review, the journal obliged me to approve a blanket statement with a broader definition of the extent of my approval. I take issue with this wording and approve the manuscript only to the extent set out in my comments.

 Observations

 1, Abstract,B ackground: In the explanation of the acronym, the wording for the 'W' doesn't follow the other wordings. Correct.

 1, Abstract, Background: There is an inconsistent use of the serial comma for enumerations in the following passage: 'herein referred to as mentees achieve their short,

 medium, and long-term research, career and professional goals' --> Either use the serial comma before 'and' and before 'or' for all enumerations/lists of two or more elements throughout the entire manuscript, or don't use it at all. I note a few more such instances. However, the entire manuscript should be rigorously corrected to follow one convention or the other, but not a mixture of both. I would suggest using a serial comma everywhere, but that is really just a personal preference.

 3: The Makerere University Research Training Programme in Infection and Immunity (MUII) programme is --> The Makerere University Research Training Programme in Infection and Immunity (MUII) is

 3: for over 10 years: It would be better to just write since when (at least the year; e.g., since April 2011). The current wording is vague.

 3: and 23 Masters fellowships. --> and 23 masters fellowships. There are a number of instances in which 'Masters' is capitalized. I would use lowercase.

 3: was the successful informal group mentorship sessions among the MUII fellows. --> I don't find a source supporting your claim that the group mentorship sessions were successful. What is the basis of this claim? What definition and operationalization of success are being used? If it is more of a personal impression by one or more of the authors, then please explain that -- and add a source. On a related note, do you explain the "-plus" part of the acronym? I suddenly realized on p. 3 that I did not understand the meaning of the "-plus" in the name of the program (even though it is part of the program name and also included in the title of your study).

 3: Masters fellows: Why is this one category of fellow capitalized? I would suggest lowercase throughout, unless you have some compelling reason. Further down, on the same page, you write "masters and PhD students."

 3: The wording with which you explain the GROW acronym is different here than in the abstract. Unless you have some specific reason (which would need to be explained), you should use a consistent wording when you explain the acronym both times. Also, see my remark about about the wording when explaining the acronym in the abstract. And who developed the GROW model? This should be stated. If the model was created by the team doing the mentoring program, this should be explained.

 3: You claim that allowing mentees to choose their mentors "is a good practice because it promotes mutual respect." This claim needs to be supported by research, and I suspect their is research on this.

 3: 2) assess the Strengths Weaknesses Opportunities Threats (SWOT) --> assess the strengths, weaknesses, opportunities, and threats (a SWOT analysis) [I don't see why using the acronym means that standard punctuation is no longer to be used; whether or not you have a comma before "and threats" depends on the decision you take for the use of the serial comma across the entire manuscript; later on, you define SWOT using lowercase words, so please standardize that]

 3: based on the principles of Plan, Do, Study, Act (PDSA); --> based on the principles of plan, do, study, and act (PDSA); [be sure to either always use lowercase in such cases unless the journal has a rule to do so with capital letters; on p. 5, you write out the SWOT abbreviation using lowercase words]

 3: SWOT and PDSA: provide sources for the methods you used. I know that the SWOT analysis is widespread and people will recognize it. However, it would be methodologically better to have a source so readers know specifically which SWOT method you applied. If you indeed came up with your own SWOT analysis, explain that and note an example of a SWOT analysis that is similar to what you did.

 3: In the first paragraph of the Methods section, I don't understand why you go back and forth between writing "MUII-plus" and "MUII." Please explain the acronym in its entirety, including the "-plus" (see my feedback above), then apply it consistently. If it is simply a matter of economy, then have one clear shift to the shorter form of the acronym, explain that choice to your readers (for example, in a brief parenthetical remark), and then consistently apply the shorter form of the acronym. However, since the acronym isn't that long and the "-plus" is even in the title of your manuscript, maybe it's best to consistently use the full acronym.

 3: In the same paragraph, you write 'utilisation' (correct in UK English, for example) and 'utilization' (correct in US English, for example). Please make sure your entire manuscript is using one English orthography. I'm guessing that the journal provides guidance about this. Please review the entire manuscript in your word processor with spell check turned on (to one English variety throughout) to make sure you have consistent spelling. This may sound trivial, but in my experience working collaboratively on texts with various authors (using various devices), the language settings get easily mixed up within one manuscript.

 3: to respondents that included masters and PhD students, mentors and leaders of the MUII programme --> to respondents that included masters and PhD students, mentors, and leaders of the MUII programme [Just one more example of what is needed if you decide to consistently use the serial comma.]

 3: The survey data included age, gender, role of respondent in the MUII-plus, duration on the MUII programme, rating experience in the mentorship programme, suggested areas of improvement of the mentorship programme, areas of knowledge or skill transfer, areas of support the respondent can render to the mentorship programme. --> The survey data included age, gender, role of respondent in the MUII-plus, duration on the MUII programme, rating experience in the mentorship programme, suggested areas of improvement of the mentorship programme, areas of knowledge or skill transfer, ***and*** areas of support the respondent can render to the mentorship programme. [Here, you sort of use a serial comma, but you omit the requisite "and" that one needs prior to the final element in your list. I just added the *** to make it easier to see the correction in this document.]

 4: key informant in-depth interviews (IDIs): This sounds like a very specific method. You should provide a source, or explain what you mean by this. What constitutes a key informant, for example? That is not immediately clear and unambiguous for your readers. Without more explanation and/or sources that provide such information, your work cannot be replicated.

 4: telephone log of the 52 active MUII-plus fellows, five MUII-plus administrators and five MUII-plus alumni --> telephone log of the 52 active MUII-plus fellows, five MUII-plus administrators, and five MUII-plus alumni [Just another example of a missing serial comma. I cannot note all such instances. The entire manuscript needs to be carefully reviewed and standardized in this respect; also for lists using "or"]

 4: "A further 12 IDIs were carried out with two MUII-plus top administration, two group leaders, and four masters’ fellows, one post-doc fellow, and three MUII-plus alumni." --> Here, for example, you DO use the serial comma. Hence, I want to reiterate what I've noted in several comments: Make a decision about this, then apply it consistently throughout the entire manuscript (including in the supplementary materials).

 4: (available as Extended data10 explored --> The closing parenthesis is missing.

 4: At the end of each interview, the researcher would re-cap the different discussion points represented by the respondents and general consensus obtained and where needed, corrections made. --> This sentence is hard to parse due to the use of a subject in the first part and then, apparently, a passive construction (and general consensus obtained ...). Please revise. Maybe write two shorter sentences to help readers better follow the process. Or, one sentence with one subject that applies for all parts of the sentence.

 4: summarizing them using matrix, --> summarizing them using a matrix, [What does it mean to "use matrix"? You mean that you used a matrix to summarize themes, perhaps? At any rate, at least an indefinite article needs to be added for consistency.]

 4: analysis.The --> analysis. The

 4: was sought for the fellows --> was sought from the fellows

 5: Masters’ projects, --> masters’ projects,

 5: Specific areas that knowledge or skills were transferred are shown in Table 2. --> Specific areas in which knowledge or skills were transferred are shown in Table 2.

 5: Masters and MUII-plus PhD fellow. --> masters and MUII-plus PhD fellow.

 5: At the lowest level in your headings, you use colons to mark the end of headings. For example: "Allows knowledge transfer and academic interaction:" The colons are not set in boldface, although the heading is in boldface. This is inconsistent. One level above that (the level using boldface + italics), the period at the end of the heading (correctly) takes the same typeface as the heading. Hence, the colons need to keep the boldface of the headings.

 5: various levels for example --> various levels, for example

 5: There is evidence in the data that the mentors on the MUII-plus mentorship programme offer their best to the mentees, resulting into long life relationships that guide the mentees through their professional and academic endeavours. --> I suggest you rewrite this statement to be more cautious in how you interpret your findings. You have not conducted a randomized trial with a properly selected waitlist group. You are looking at a small number of responses from a few participants. One must keep in mind that satisfaction surveys of program participants can reflect the characteristics of the people who joined a program rather than the effects generated by the actual program offerings. Also, this remark (which I think needs to be reformulated to not over-interpret your findings) probably belongs in the discussion section rather than directly in the results.

 5: resulting into long life relationships --> resulting in lifelong relationships [I'm not familiar with 'long life relationships']

 5: visited me at my University where I was --> visited me at my university where I was [I realize this is a transcript. As such, it does not need to follow prescriptive grammar rules and the like. However, as it is a transcribed realization of spoken language, the orthography should at least be standardized. I see no clear reason my "University" should be capitalized.]

 5: Voices extracts from the alumni --> Do you mean "Voices extracted form the alumni" perhaps? Or maybe "Voice extracts from the alumni"?

 5: Many of the mentees that were paired --> Many of the mentees who were paired

 7: There is an need --> There is a need

 7: on what kind of motivation that should be given to a mentor: --> on the kind of motivation that should be given to a mentor:

 7: some said that a mentor shouldn’t be --> Some said that a mentor should not be [I would avoid contractions in formal writing. Regarding capitalization after a colon. The APA7 rule on this, for example, is that you use a capital letter after a colon when a complete sentence follows, which is the case here. Maybe the journal has a different rule in its house style. Please check the rule, then re-check all such instances in the manuscript.]

 7: while the mentee maybe motivated by successfully finishing their academic programmes and are awarded their masters, PhDs and post-docs. --> while the mentee may be motivated by successfully finishing their academic programmes and earning their masters, PhDs, and post-docs.

 7: The programme has been cited as one that has produced very good scientists that have gone ahead to impact society in various ways. The programme boasts of being a hub through which a number of fellows have had opportunities to carry out cutting-edge research which has helped them earn advanced academic degrees. --> I have two points/questions here: First, where was the program cited in this way? Do you mean cited in the interviews with the respondents in your study? Or do you literally mean cited in other publications? Please revise to make that clear. If you literally mean citations, then these need to be provided. Second, "The programme boasts of ..." seems out of place here. You are summarizing findings of you interviews, correct? Then it is confusing to write of what the "program" says (boasts) about itself. That is confusing, especially since the passage is followed by a quote from a respondent interview. I'm not sure that the statement about "The programme boasts ..." make sense here. Please review this and improve the consistency of your description of the study findings at this point.

 7: for the University departments where --> for the university departments where

 8: “….about this --> “… about this --> I'm not sure you really need any ellipses when featuring excerpts from the transcripts. It is clear from the context that you are showing excerpts. If you want to add ellipses, then use one set of three dots with a space after them.

 8 and before: The dashes with which you prepend the codes of the quoted respondents are inconsistent. You use a hyphen in some cases (e.g., "-Executive 1-KI" or "-FGD-R2") and in some cases even an em-dash(—FGD-R8). Please review all attributions after all quotes from your transcripts and provide a uniform system and style for such attributions.

 8: especially from University teaching staff --> especially from university teaching staff [I am not going to report this problem any more. Double-check the use of capitalization throughout the manuscript. I don't see the reason for capitalizing the U in university. Even if it refers to the staff of the noted university, it does not seem like a usual use of capitalization.]

 8: The fellow’s meetings offer --> The fellows’ meetings offer [you mean possessive plural, I assume?]

 9: While good mentees must have an adaptable character, be self-directed and recognise generational differences24,25. --> This is an incomplete sentence. It makes sense, but the wording should be changed to make a grammatically complete sentence. Either you can combine it with the previous sentence (..., while good mentees ...) or you can revise it to work as a stand-alone sentence (for example: "At the same time, mentees must have an ...).

 9: without inappropriate gender conflicts -->  I think I understand what you mean. However, the wording is a bit strange, because it would imply that there are possibly also 'appropriate' gender conflicts. You should revise this remark.

 9: There was adequate research support to develop research ideas or problem solving, and guidance25. --> It is hard to parse your wording here. Revise the sentence, perhaps like this: The program provided adequate research support for developing research ideas and solving problems as well as sufficient guidance25.

 9: which have in one way impacted the lives of the fellows. --> which have impacted the lives of the fellows in one way or another. [Is that perhaps what you mean here?]

 9: In this vain, --> In this vein

 9: the different mentorship styles --> Based on my understanding of the literature, I would suggest you refer here to mentoring 'roles' rather than 'styles.' It is more concrete and better aligned with existing research. Either way, you should cite at least one source that outlines 'mentoring roles' (my preference) or what you understand as 'mentorship styles.' Considering its title, the publication you currently cite (30 in your references) appears to refer to 'roles' not 'styles.'

 9: should be based on the Martin Buber’s theory of --> should be based on Martin Buber’s theory of [or even just 'Buber's theory of ...' but not 'the ...']

 9: nature of mentee–mentor relationship --> nature of the mentee–mentor relationship

 9: between formal mentor role as the advisor with the informal role as the friend --> between the formal mentor role as the advisor with the informal role as the friend

 9: Multiple roles might result into role conflict --> Multiple roles might result in role conflict

 9: Mentors’ busy schedules has been reported in other capacity-building programmes. --> Mentors’ busy schedules have been reported in other capacity-building programmes.

 9: This study, however, was not able to access such competencies. --> The wording isn't clearly incorrect. However, it is a bit unusual. You might want to use "assess" rather than "access," as in: This study, however, was not able to assess such competencies.

 9: their work life challenges --> Either write "their work–life challenges" with an en-dash (–) if you mean something along the lines of 'challenges in balancing work demands against demands of life more generally' or write "their work-life challenges" with a hyphen (-) if you means challenges posed by work life. As it is (with neither an en-dash nor a hyphen), the phrase is ambiguous.

 9: They too usually lack research mentorship33. --> They also usually lack research mentorship33.

 9: Work life pressure --> Same point here: Either write "Work–life pressure" or "Work-life pressure" depending on your intended meaning.

 9: mentee-mentor agreements --> mentee–mentor agreements [The authors have been very good about using an en-dash when writing 'mentee–mentor' followed by a noun, which is correct; this one slipped through and needs to be fixed]

 10: capacity building programmes --> capacity-building programmes

 10: ensures transfer of knowledge and skills --> ensures a transfer of knowledge and skills

 10: to foster continuity to the programme. --> to foster continuity in the programme.

 10: to know what the difficulties where for the mentors --> to know what the difficulties were for the mentors

 10: and if there was a way to make the mentorship relationship a win–win for both the mentor and the mentee. --> and whether there was a way to make the mentorship relationship a win–win for both the mentor and the mentee. [In formal writing 'whether' should be used instead of 'if' in the sense in which you are using it here]

 10: seven have been part --> seven had been part

 10: the benefits of cross national / institution mentorship. --> This is currently ambiguous. Do you mean 'cross-national and/or cross-institution mentorship,' for example, or do you mean 'cross-national and/or institution mentorship'? You probably mean the first option. In that case, write it out to be precise: 'cross-national and/or cross-institution mentorship'

 10: There might be some slight bias with the alumni since the formal MUII-plus mentorship programme started after they left and therefore might have introduced some social desirability bias. --> It is good you point this out. I suspect this can be the case. However, I don't see any basis for qualifying this with "some slight." As far as I can think it through, more research would be needed to estimate the strength of any such bias. For that reason, I suggest you cut out the speculation here and write, for example: "There might be bias with the alumni since the formal MUII-plus mentorship programme started after they left, which might have introduced some social desirability bias." A more circumspect wording here strengthens your paper!

 10: However, this was managed by the study team reviewing and agreeing on the findings. --> I don't find this convincing, because it is too vague. The best option is probably to simply cut this sentence. If you really want to go into the steps you took to address this possible bias, then you need to explain these to your readers. For example: However, we attempted to address this possible bias by ...

 10: Also, the SWOT analysis could have assessed the mentorship programme at three levels namely institutional, --> Also, the SWOT analysis could have assessed the mentorship programme at three levels, namely, institutional,

 10: and it is important for the leadership to know that the respondents believe in the programme and are ready to accord it all the necessary support. --> I am not convinced by the second of the two claims being made in this sentence. According to what I learned from reading your manuscript, mentor respondents readily admitted that they couldn't provide sufficient mentoring due to the time constraints they were facing in their lives. That finding is common in mentoring programs and an important finding of your study. By claiming at the start of your concluding summary that you found out that respondents were ready to accord the mentoring program "all the necessary support," you overlook that finding. Readers may take away an over-simplified message and forget the other finding, which is, in my view, much more important. At this point, you don't need to 'sell' your study or your program!

 10: still enjoys support of both the mentors, mentees and alumni --> still enjoys support of the mentors, mentees and alumni [the 'both' is confusing here because you have a list of three things. If you really want to keep the 'both,' then you need to revise the wording, for example: still enjoys support of both the mentors and the mentees as well as of the alumni]

 10: Employ several opportunities for speed mentorship to avoid fatigue from long relationships39 --> I do not know the study you cite. However, in looking at its title, it sounds to me like that is about helping mentors and mentees to quickly/efficiently find good matches. Long-term relationships are essential for effective mentoring. It is unrealistic to think that short-term mentoring is a good solution. Should you quickly re-match when a relationship doesn't work out? Yes, definitely! Can you hope to overcome resource issues related to insufficient mentor time by working with short-term mentoring? I am very skeptical of that. Please rethink recommendation 2. Maybe it's just the wording that needs to be revised to avoid an implication you don't wish to make.

 10: top down directional mentorship to bottom up approach --> top-down directional mentorship to a bottom-up approach

 10: also include check lists --> also include checklists

Is the work clearly and accurately presented and does it cite the current literature?

Partly

If applicable, is the statistical analysis and its interpretation appropriate?

Not applicable

Are all the source data underlying the results available to ensure full reproducibility?

Yes

Is the study design appropriate and is the work technically sound?

Yes

Are the conclusions drawn adequately supported by the results?

Yes

Are sufficient details of methods and analysis provided to allow replication by others?

Yes

Reviewer Expertise:

Education

I confirm that I have read this submission and believe that I have an appropriate level of expertise to confirm that it is of an acceptable scientific standard.

# Reviewer report: Reviewer response for version 2

**DOI:** 10.21956/aasopenres.14323.r31019

**Published:** 2024-05-09

**Authors:** Tracy Alexis Kakyo

**Affiliations:** 1 Flinders University, Adelaide, Australia

Well done to the authors for the work they are doing to incorporate institutionalised mentoring in career development of the health scientists in Uganda. Overall, the manuscript is clear and well written. My comments serve to improve adaptability of the program by other scientist but also ease of follow by the reader to track how the theory used was incorporated in the design of the program and in the analysis of the findings.

 The authors describe the progression of the MUII program over the 10 years. The program has evolved over the time in implementation using two models: MUII (used informal approaches to mentoring) and MUII-Plus (the formalised program).  My question is which of the two models is being evaluated in this paper. The authors move alternately between MUII and MUII-Plus.  

 The formal mentoring programme describes the matching processes from the mentor and mentee however the details that are essential are missing. How was GROW incorporated in the program, how often did the mentors and mentees meet, how long was the mentoring relationship, etc. This would enable adaptation of this innovation to other setting with similar context.

 In the background section, the authors report of 68 fellows while in the results section the number reported is 52.

 There is overlap in the presentation of findings and there is not enough explanation of these sub-themes to justify the overlap. For example, ‘ *busy schedule of mentors’* is both a weakness and a threat. This overlap is not adequately explained why this is considered both a threat and a weakness.

 The same argument applies to other findings listed under “ *Factors leading to a sustainable and successful…” *a critical look they all can be placed under SWOT framework as subthemes.

 The authors also mention “ *Other achievements*”, this label gives the impression that main achievements had been outlined somewhere within the manuscript, which does not appear to be the case.

 Table 3 is not cited in text. I am also curious why it centres on only the Key Informant interviews as opposed to entire aggregation of all the three data sets. The data sets can be varied but they all talk about the same thing

 Well done to the authors for the work they are doing to incorporate institutionalised mentoring in career development of the health scientists in Uganda. Overall, the manuscript is clear and well written. My comments serve to improve adaptability of the program by other scientist but also ease of follow by the reader to track how the theory used was incorporated in the design of the program and in the analysis of the findings.

 The authors describe the progression of the MUII program over the 10 years. The program has evolved over the time in implementation using two models: MUII (used informal approaches to mentoring) and MUII-Plus (the formalised program).  My question is which of the two models is being evaluated in this paper. The authors move alternately between MUII and MUII-Plus.  

 The formal mentoring programme describes the matching processes from the mentor and mentee however the details that are essential are missing. How was GROW incorporated in the program, how often did the mentors and mentees meet, how long was the mentoring relationship, etc. This would enable adaptation of this innovation to other setting with similar context.

 In the background section, the authors report of 68 fellows while in the results section the number reported is 52.

 There is overlap in the presentation of findings and there is not enough explanation of these sub-themes to justify the overlap. For example, ‘ *busy schedule of mentors’* is both a weakness and a threat. This overlap is not adequately explained why this is considered both a threat and a weakness.

 The same argument applies to other findings listed under “ *Factors leading to a sustainable and successful…” *a critical look they all can be placed under SWOT framework as subthemes.

 The authors also mention “ *Other achievements*”, this label gives the impression that main achievements had been outlined somewhere within the manuscript, which does not appear to be the case.

 Table 3 is not cited in text. I am also curious why it centres on only the Key Informant interviews as opposed to entire aggregation of all the three data sets. The data sets can be varied but they all talk about the same thing

Is the work clearly and accurately presented and does it cite the current literature?

Yes

If applicable, is the statistical analysis and its interpretation appropriate?

Yes

Are all the source data underlying the results available to ensure full reproducibility?

No source data required

Is the study design appropriate and is the work technically sound?

Yes

Are the conclusions drawn adequately supported by the results?

Yes

Are sufficient details of methods and analysis provided to allow replication by others?

Partly

Reviewer Expertise:

Mentoring, professional development, nursing education

I confirm that I have read this submission and believe that I have an appropriate level of expertise to confirm that it is of an acceptable scientific standard, however I have significant reservations, as outlined above.

# Reviewer report: Reviewer response for version 1

**DOI:** 10.21956/aasopenres.14187.r28008

**Published:** 2020-12-08

**Authors:** Joseph R. Zunt

**Affiliations:** 1 Department of Internal Medicine, University of Washington School of Medicine, Seattle, WA, USA

Mentoring is an essential component of all research training programs. Dr. Andira Biraro and colleagues detail their SWOT analysis of the MUII-plus mentorship program in Uganda. One of the factors they identified that was a weakness and a threat was lack of time for mentoring - which is a problem throughout the academic world - regardless of the location; I think the authors should include mention of this in the abstract under Results (no Weaknesses were included). 

 More detail regarding the structure of mentoring would have been welcome. Were all mentees mentored by both Ugandan and UK mentors? If not, were mentees with mentors from both countries more satisfied with their mentoring experience?

 The authors included paragraphs in various sections of the manuscript regarding lack of time, rewarding mentors, engaging alumni, training mentors, defining expectations - I would have found it very useful to have either combined all of these factors into a specific section on mentoring or into an additional table.

 Under conclusions, I would have liked to see more recommendations regarding increasing recognition for mentoring, as I don't think that recognizing a mentor as a "coach" or "certified" mentor, without additional recognition from the institution - either percent effort or FTE provided for mentoring - would result in increased mentor satisfaction or increased availability to mentor.

Minor grammatical errors:

- Page 3: "ono-to-one" should be "one-to-one".
- Page 7: "no tangible" should be "intangible" and, in following sentence, "maybe" should be "may be".

Tables: 

- Table 1 doesn't add much to manuscript.
- Tables 2 and 3 contain important information and could be combined.

Is the work clearly and accurately presented and does it cite the current literature?

Yes

If applicable, is the statistical analysis and its interpretation appropriate?

Yes

Are all the source data underlying the results available to ensure full reproducibility?

Yes

Is the study design appropriate and is the work technically sound?

Yes

Are the conclusions drawn adequately supported by the results?

Yes

Are sufficient details of methods and analysis provided to allow replication by others?

Yes

Reviewer Expertise:

Neurology, global health, epidemiology, infectious diseases

I confirm that I have read this submission and believe that I have an appropriate level of expertise to confirm that it is of an acceptable scientific standard.

# Response

**Published:** 2020-12-10

**Authors:** Irene Andia Biraro

**Affiliations:** Makerere University, Uganda

**REBUTTAL LETTER FOR MANUSCRIPT TITLED: “** **Analysis of the MUII-plus Mentorship Programme: Reflections of Fellows’ Experiences and Lessons for Other Programmes”**

**Query 1:** Mentoring is an essential component of all research training programs. Dr. Andira Biraro and colleagues detail their SWOT analysis of the MUII-plus mentorship program in Uganda. One of the factors they identified that was a weakness and a threat was lack of time for mentoring - which is a problem throughout the academic world - regardless of the location; I think the authors should include mention of this in the abstract under Results (no Weaknesses were included). 

**Response 1: Thank you for this comment. This has been added in the abstract** **in the revised version on page 2.**

***“*** ***The biggest weakness that also presented as a t*** ***hreat to the mentorship programme was the busy schedule of mentors.*** ***”***

**Query 2: **More detail regarding the structure of mentoring would have been welcome. Were all mentees mentored by both Ugandan and UK mentors? If not, were mentees with mentors from both countries more satisfied with their mentoring experience?

**Response 2: Thank you for this comment. We did not assess how many mentees had both Ugandan and International mentors and therefore we are not able to assess the benefits of such mentorship. This has been added to the limitations in the discussion section on page 16 of the revised version.**

***“*** ***We also made a major omission of not asking the mentees how many had both Ugandan and International mentors, and therefore we unable to assess the benefits of cross national / institution mentorship.*** ***”***

**Query 3:** The authors included paragraphs in various sections of the manuscript regarding lack of time, rewarding mentors, engaging alumni, training mentors, defining expectations - I would have found it very useful to have either combined all of these factors into a specific section on mentoring or into an additional table.

***Response 3: Thank you for this comment. The nature of the SWOT analysis of the mentorship programme led to such repetition that we were unable to merge all into one section without distorting the SWOT structure.  Some themes did appear in more than one area such a busy nature of mentors.***

**Query 4:** Under conclusions, I would have liked to see more recommendations regarding increasing recognition for mentoring, as I don't think that recognizing a mentor as a "coach" or "certified" mentor, without additional recognition from the institution - either percent effort or FTE provided for mentoring - would result in increased mentor satisfaction or increased availability to mentor.

**Response 4: Thank you for you observation. A better recommendation has been added to the conclusion number six on page 17 in the revised version.**

***“*** ***Advocate for more tangible recognition of mentors especially in academic institutions that can be considered towards promotion or career enhancement. In addition establish coach mentors or certified mentors [39, 40] as a another form of recognition for mentors.*** ***”***

Minor grammatical errors:

**Query 5:** Page 3: "ono-to-one" should be "one-to-one".

**Response 5: Thank you. We have corrected this typographical error.**

**Query 6:** Page 7: "no tangible" should be "intangible" and, in following sentence, "maybe" should be "may be".

**Response 6: This has been addressed and corrected.**

Tables:

**Query 7:** Table 1 doesn't add much to manuscript.

**Response 7: Thank you for this comment. We however were advised to try and show some description of the participants to help contextualise some aspects of the paper.**

**Query 8:** Tables 2 and 3 contain important information and could be combined.

**Response 8: Thank you for this very good suggestion. We have combined tables 2 and 3 into one table.**

# Response

**Published:** 2021-02-22

**Authors:** Irene Andia Biraro

**Affiliations:** Makerere University, Uganda

**REBUTTAL LETTER TO REVIEWER TWO FOR MANUSCRIPT TITLED: “Analysis of the MUII-plus Mentorship Programme: Reflections of Fellows’ Experiences and Lessons for Other Programmes”**

**Query 1:** Mentoring is an essential component of all research training programs. Dr. Andira Biraro and colleagues detail their SWOT analysis of the MUII-plus mentorship program in Uganda. One of the factors they identified that was a weakness and a threat was lack of time for mentoring - which is a problem throughout the academic world - regardless of the location; I think the authors should include mention of this in the abstract under Results (no Weaknesses were included). 

**Response 1: Thank you for this comment. This has been added in the abstract in the revised version on page 2.**

***“The biggest weakness that also presented as a threat to the mentorship programme was the busy schedule of mentors.”***

**Query 2: **More detail regarding the structure of mentoring would have been welcome. Were all mentees mentored by both Ugandan and UK mentors? If not, were mentees with mentors from both countries more satisfied with their mentoring experience?

**Response 2: Thank you for this comment. We did not assess how many mentees had both Ugandan and International mentors and therefore we are not able to assess the benefits of such mentorship. This has been added to the limitations in the discussion section on page 16 of the revised version.**

***“We also made a major omission of not asking the mentees how many had both Ugandan and International mentors, and therefore we unable to assess the benefits of cross national / institution mentorship.”***

**Query 3:** The authors included paragraphs in various sections of the manuscript regarding lack of time, rewarding mentors, engaging alumni, training mentors, defining expectations - I would have found it very useful to have either combined all of these factors into a specific section on mentoring or into an additional table.

***Response 3: Thank you for this comment. The nature of the SWOT analysis of the mentorship programme led to such repetition that we were unable to merge all into one section without distorting the SWOT structure.  Some themes did appear in more than one area such a busy nature of mentors.***

**Query 4:** Under conclusions, I would have liked to see more recommendations regarding increasing recognition for mentoring, as I don't think that recognizing a mentor as a "coach" or "certified" mentor, without additional recognition from the institution - either percent effort or FTE provided for mentoring - would result in increased mentor satisfaction or increased availability to mentor.

**Response 4: Thank you for you observation. A better recommendation has been added to the conclusion number six on page 17 in the revised version.**

***“Advocate for more tangible recognition of mentors especially in academic institutions that can be considered towards promotion or career enhancement. In addition establish coach mentors or certified mentors as a another form of recognition for mentors.”***

Minor grammatical errors:

**Query 5:** Page 3: "ono-to-one" should be "one-to-one".

**Response 5: Thank you. We have corrected this typographical error.**

**Query 6:** Page 7: "no tangible" should be "intangible" and, in following sentence, "maybe" should be "may be".

**Response 6: This has been addressed and corrected.**

Tables:

**Query 7:** Table 1 doesn't add much to manuscript.

**Response 7: Thank you for this comment. We however were advised to try and show some description of the participants to help contextualise some aspects of the paper.**

**Query 8:** Tables 2 and 3 contain important information and could be combined.

**Response 8: Thank you for this very good suggestion. We have combined tables 2 and 3 into one table as the current table 2 on page 24.**

# Reviewer report: Reviewer response for version 1

**DOI:** 10.21956/aasopenres.14187.r27970

**Published:** 2020-11-30

**Authors:** Emilia Noormahomed

**Affiliations:** 1 Department of Medicine, Division of Infectious Diseases and Global Public Health, University of California, San Diego, San Diego, CA, USA; 2 Eduardo Mondlane University, Maputo, Mozambique

In general, the manuscript structure is well organized and written clearly.

 Though it is not clearly stated in the abstract, this manuscript seems to want to describe strategies and innovations developed under the MUII-plus mentorship program, reflexions of fellows and lessons learned, as a way build to research capacity, and career development. It ends by drawing conclusions and recommendations that can be applied or adapted to other settings which lack human resources with didactic and research skills for mentorship and ways to make the program goals sustainable.  

 So, my first recommendation to the authors is to clearly state the aims of the manuscript in the abstract. In addition, I recommend the authors to correct the title by eliminating the acronyms **MUII-plus** and instead spell out all words. The same recommendation is applied for the abstract, where the authors should spell out the acronyms MUII the first time they write it.

 Mentorship in low-in-come countries is a topic of great interest in global health, as many institutions in this setting do not have an institutionalized system of mentorship or if present are deficient, due to several reasons. Amongst the various weakness for the success of a mentorship system, the busy schedule of mentors has been pointed as one factor that hamper the success of mentorship. However, we did not see any comment about the overall number of fellows admitted, their quality, and possible drop outs. It would be important to clarify, and try to establish any relation with some possible mentor’s lake of commitment. This is to say that the attitude of the mentees may also influence the attitude of the mentor and motivation to continue to act as a mentor.

 The methodology they used is appropriately described and reproducible. The results are well stated and appropriately discussed.

 Taking into account the importance of the topic they address for future initiatives, lessons learned, and recommendations addressed, I would strongly recommend the acceptance of this manuscript for indexing, after the authors have addressed the minor revisions I propose.

Is the work clearly and accurately presented and does it cite the current literature?

Yes

If applicable, is the statistical analysis and its interpretation appropriate?

Yes

Are all the source data underlying the results available to ensure full reproducibility?

Yes

Is the study design appropriate and is the work technically sound?

Yes

Are the conclusions drawn adequately supported by the results?

Yes

Are sufficient details of methods and analysis provided to allow replication by others?

Yes

Reviewer Expertise:

NA

I confirm that I have read this submission and believe that I have an appropriate level of expertise to confirm that it is of an acceptable scientific standard, however I have significant reservations, as outlined above.

# Response

**Published:** 2020-12-10

**Authors:** Irene Andia Biraro

**Affiliations:** Makerere University, Uganda

**Query 1:** So, my first recommendation to the authors is to clearly state the aims of the manuscript in the abstract. In addition, I recommend the authors to correct the title by eliminating the acronyms **MUII-plus** and instead spell out all words. The same recommendation is applied for the abstract, where the authors should spell out the acronyms MUII the first time they write it.

**Response 1: Thank you for this comment. MUII-plus has been written in full in the both the title and the abstract of the revised manuscript.**

**“Analysis of the Makerere University Research Training Programme in Infection and Immunity (MUII) Mentorship Programme: Reflections of Fellows’ Experiences and Lessons for Other Programmes”**

**Query 2:** Mentorship in low-in-come countries is a topic of great interest in global health, as many institutions in this setting do not have an institutionalized system of mentorship or if present are deficient, due to several reasons. Amongst the various weakness for the success of a mentorship system, the busy schedule of mentors has been pointed as one factor that hamper the success of mentorship. However, we did not see any comment about the overall number of fellows admitted, their quality, and possible drop outs. It would be important to clarify, and try to establish any relation with some possible mentor’s lake of commitment. This is to say that the attitude of the mentees may also influence the attitude of the mentor and motivation to continue to act as a mentor.

**Response 2: Thank you for this important comment. The programme has 68 fellows and all these have been part of the mentorship programme in one way or another. We did not assess how many dropped off the mentorship programme at the time of the survey but we can assume that many had since we had a poor response rate to our survey. We further assume that one of the factors of the poor participation is busy mentors. However, we were not able to demonstrate this in this paper. We also hardly got responses from mentors. We attempted to address this under the limitation section of the discussion.**

# Response

**Published:** 2021-02-22

**Authors:** Irene Andia Biraro

**Affiliations:** Makerere University, Uganda

**Author: REBUTTAL LETTER TO REVIEWER ONE FOR MANUSCRIPT TITLED: “Analysis of the MUII-plus Mentorship Programme: Reflections of Fellows’ Experiences and Lessons for Other Programmes”**

**Query 1:** So, my first recommendation to the authors is to clearly state the aims of the manuscript in the abstract. In addition, I recommend the authors to correct the title by eliminating the acronyms **MUII-plus** and instead spell out all words. The same recommendation is applied for the abstract, where the authors should spell out the acronyms MUII the first time they write it.

**Response 1: Thank you for this comment. MUII-plus has been written in full in the both the title and the abstract of the revised manuscript.**

**“Analysis of the Makerere University Research Training Programme in Infection and Immunity (MUII) Mentorship Programme: Reflections of Fellows’ Experiences and Lessons for Other Programmes”**

**Query 2:** Mentorship in low-in-come countries is a topic of great interest in global health, as many institutions in this setting do not have an institutionalized system of mentorship or if present are deficient, due to several reasons. Amongst the various weakness for the success of a mentorship system, the busy schedule of mentors has been pointed as one factor that hamper the success of mentorship. However, we did not see any comment about the overall number of fellows admitted, their quality, and possible drop outs. It would be important to clarify, and try to establish any relation with some possible mentor’s lake of commitment. This is to say that the attitude of the mentees may also influence the attitude of the mentor and motivation to continue to act as a mentor.

**Response 2: Thank you for this important comment. The programme has 68 fellows and all these have been part of the mentorship programme in one way or another. We did not assess how many dropped off the mentorship programme at the time of the survey but we can assume that many had since we had a poor response rate to our survey. We further assume that one of the factors of the poor participation is busy mentors. However, we were not able to demonstrate this in this paper. We also hardly got responses from mentors. We attempted to address this under the limitation section of the discussion on page 15 of the revised manuscript.**
